# Sexual conflict in a changing environment

**DOI:** 10.1111/brv.12728

**Published:** 2021-05-07

**Authors:** Agata Plesnar‐Bielak, Aleksandra Łukasiewicz

**Affiliations:** ^1^ Institute of Environmental Sciences, Faculty of Biology Jagiellonian University ul. Gronostajowa 7 30‐387 Kraków Poland; ^2^ Department of Environmental and Biological Sciences University of Eastern Finland PO Box 111 80101 Joensuu Finland; ^3^ Evolutionary Biology Group, Faculty of Biology Adam Mickiewicz University ul. Uniwersytetu Poznańskiego 6 61‐614 Poznań Poland

**Keywords:** sexual conflict, sexual selection, sexual antagonism, male harm, female resistance, sex‐specific selection, environmental change, stress, adaptation, gender load

## Abstract

Sexual conflict has extremely important consequences for various evolutionary processes including its effect on local adaptation and extinction probability during environmental change. The awareness that the intensity and dynamics of sexual conflict is highly dependent on the ecological setting of a population has grown in recent years, but much work is yet to be done. Here, we review progress in our understanding of the ecology of sexual conflict and how the environmental sensitivity of such conflict feeds back into population adaptivity and demography, which, in turn, determine a population's chances of surviving a sudden environmental change. We link two possible forms of sexual conflict – intralocus and interlocus sexual conflict – in an environmental context and identify major gaps in our knowledge. These include sexual conflict responses to fluctuating and oscillating environmental changes and its influence on the interplay between interlocus and intralocus sexual conflict, among others. We also highlight the need to move our investigations into more natural settings and to investigate sexual conflict dynamics in wild populations.

## I. INTRODUCTION

Sexual conflict, resulting from differential evolutionary interests and reproductive strategies of the sexes (Parker, 1979; Chippindale, Gibson & Rice, 2001; Chapman *et al*., 2003), is nearly inextricably associated with sexual selection (Arnqvist & Rowe, 2005; Connallon & Clark, 2014): some degree of sexual conflict is present in almost all sexually reproducing species. It can occur either within a locus (intralocus sexual conflict; IASC, see Table 1) or between two loci (interlocus sexual conflict; IESC, see Table 1). Under IASC, the direction of selection on a shared trait depends on the sex in which this trait is expressed (Chippindale *et al*., 2001; Bonduriansky & Chenoweth, 2009). A well‐known example of such conflict is height in Western human populations, in which reproductive success is maximized for average‐height men but shorter women (Stulp *et al*., 2012). IESC, on the contrary, is conflict over the outcome of reproductive interactions. Under IESC, selection on a trait in one sex may result in the evolution of another trait in the opposite sex (Parker, 1979; Chapman *et al*., 2003; Arnqvist & Rowe, 2005). For example, optimal mating rate is usually substantially higher for males than for females [but see Bro‐Jørgensen (2007) for an example of ‘reversed conflict’], generating sexual conflict that leads to the evolution of male traits coercing females into copulation and of female traits defending from this coercion.

**Table 1:** Glossary of the most important terminology

Term	Definition
Sexual conflict	Conflict resulting from differential evolutionary interests and strategies of the sexes
Intralocus sexual conflict (IASC)	Sexual conflict in which selection on a shared allelic trait acts in opposite directions in males and females, displacing one or both sexes from their evolutionary optima
Interlocus sexual conflict (IESC)	Sexual conflict that arises from a sex‐specific difference in optimal outcome of a male–female interaction, so that a trait for which male and female evolutionary optima differ depends on different loci, coding for traits manipulating outcomes of reproductive interactions
Intersexual genetic correlation for fitness (r_MF_)	Commonly used measure of intralocus sexual conflict intensity; the ratio of the additive genetic covariance for fitness between the sexes to the geometric mean of male and female additive genetic variance for fitness
Gender load	Reduction of the mean fitness of a population caused by sexual conflict
Sexually antagonistic coevolution (SAC)	An evolutionary process where traits under IESC change over time counteracting changes in traits in the opposite sex to maximize reproductive success
Harm	Reduction in fitness resulting from adaptations in the other sex to manipulate a trait under IESC that increase their reproductive success
Resistance	The ability to counter adaptations in the other sex to manipulate a trait under IESC
Tragedy of the commons	A term originating from economics describing a situation where individuals acting in their self‐interest overexploit resources available in the system and lead to resource depletion. In a sexual conflict context, tragedy of the commons is used to illustrate that male adaptations that increase their fitness can result in a reduction in population fitness and viability

Sexual conflict is not only ubiquitous, but also plays a role in shaping various evolutionary processes including, for example, population divergence and speciation (Gavrilets & Hayashi, 2005; Gavrilets, 2014), maintenance of genetic variation (Rice & Chippindale, 2001), evolution of aging and senescence (Promislow, 2003; Bonduriansky *et al*., 2008; Fricke *et al*., 2013), and regulation of gene expression (Ellegren & Parsch, 2007). Given the breadth of its effects, it is not surprising that sexual conflict and its consequences have been studied extensively in recent decades. Importantly, however, the role of ecological factors in shaping both IASC and IESC patterns and modulating their intensity has been acknowledged only recently (e.g. Rowe *et al*., 1994; Cornwallis & Uller, 2010; Fricke, Bretman & Chapman, 2010*b*; Arbuthnott *et al*., 2014*b*; Perry, Garroway & Rowe, 2017; Svensson, 2019). The role of ecology in shaping sexual conflict patterns is especially relevant as sexual interactions, reproductive roles and sex‐specific traits do not evolve in an ecological vacuum. Quite the contrary, costs and benefits of all reproductive strategies and investment decisions should always depend on ecological factors. As a consequence, the evolution of all traits under sexual selection, including those under conflict, should to some extent be influenced by ecology. The same is true for many life‐history traits, which are often associated with intralocus conflict between the sexes (Wedell *et al*., 2006; Long & Rice, 2007; Lewis, Wedell & Hunt, 2011; Berg & Maklakov, 2012; Berger *et al*., 2014).

Currently, a considerable effort is being made to understand better the relationship between ecology and sexual conflict, with the number of studies in this exciting and important field growing rapidly. In the present review, we summarize current knowledge on how the environment modifies sexual conflict and discuss implications of this work for evolutionary biology research, including studies on local adaptation. We also highlight areas where more work is needed and identify perspectives for future research. Since IESC and IASC differ in mechanisms, evolutionary dynamics and evolutionary outcomes, and might be differentially influenced by environment (see Van Doorn, 2009; Schenkel *et al*., 2018), a separate subsection is dedicated to each of these types of conflict. The sections differ in structure, reflecting differences in the nature of environmental impacts on the two conflict forms. Specifically, ecological factors are predicted to modify the intensity of IASC, while ecological influences on IESC may be more complex, because ecology can directly or indirectly impact different aspects of conflict dynamics. In addition, there is no straightforward measure of IESC intensity (see Arnqvist & Rowe, 2005). Consequently, for IASC we focus on discussing how ecology influences conflict levels, while for IESC we concentrate on environmental effects on conflict dynamics and the interplay between different male and female traits and behaviours as well as environmental plasticity in conflict‐related traits. We also discuss the environmental sensitivity of sexual conflict in the context of environmental change and adaptation.

We have to acknowledge that in many cases the genetic basis of traits under sexual conflict is unknown, so that the distinction between IASC and IESC is sometimes uncertain and based on indirect evidence (but see Schenkel *et al*., 2018). Moreover, IASC and IESC may be linked in two not mutually exclusive ways. First, both forms of sexual conflict may concern the same traits involved in reproduction (Gibson, Chippindale & Rice, 2002; Andrés & Morrow, 2003; Rice *et al*., 2005; Innocenti & Morrow, 2010; Stewart, Pischedda & Rice, 2010). For many reproductive traits, selection pressures resulting from interactions with the opposite sex (IESC) can affect sex‐specific optima, leading to IASC (see Abbott, 2011). Second, complex genetic bases of reproductive traits associated with IESC may involve pleiotropic effects in both sexes, and thus be exposed to IASC (Pennell & Morrow, 2013). Recent theory predicts that interaction between IASC and IESC may have different outcomes for antagonistic coevolution (Pennell *et al*., 2016), depending on the degree of sex‐limited expression of mating traits and their distance from evolutionary optima. IASC can stabilize arms races between the sexes counteracting escalation of IESC, but may also prevent populations from reaching equilibrium, causing populations to enter a never‐ending cycle of arms race and accumulation of IASC followed by periods of IASC resolution that fuel another arms race (Pennell *et al*., 2016). Thus, in the final part of this review, we link these types of conflict, discuss how their interaction is shaped by environment and list outstanding questions that need to be answered to understand the role of ecology in shaping conflict dynamics and how it feeds back into evolutionary processes.

## II. INTRALOCUS SEXUAL CONFLICT AND ENVIRONMENT

### (1) Theory

**Fig 1:**
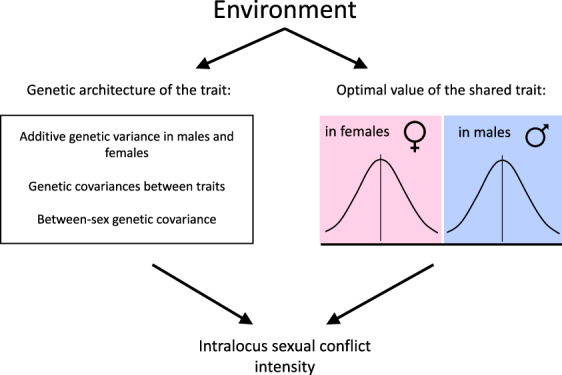
Schematic representation of the ways environmental factors can modify intralocus sexual conflict (IASC) intensity.

IASC results from sexually dimorphic evolutionary optima for traits whose genetic background is shared by the sexes (i.e. there is a positive between‐sex genetic correlation for these traits; see Table 1). As a consequence, selection on a trait under IASC acts in an opposite direction in males and females, generating a negative fitness correlation between the sexes. Environmental conditions may shape optimal trait values for the sexes differentially, so that conflict might be apparent under some conditions, but reduced or absent under others (Fig. 1).

**Fig 2:**
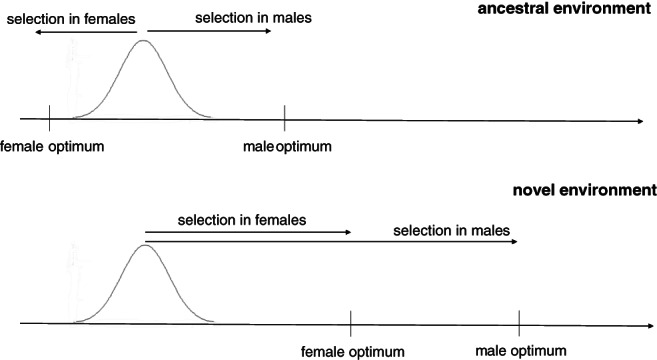
Schematic representation of the distribution of a possible shared trait and its optimum in the sexes in ancestral (upper panel) and novel (lower panel) environments.

A simple expectation is that IASC should be most intense in an environment to which a population is well adapted so that both sexes are relatively close to their evolutionary optima. This is because most mutations with sex concordant effects on fitness should either be fixed (if beneficial to both sexes) or purged (if maladaptive) in such environments, while sexually antagonistic alleles may be maintained (Rice & Chippindale, 2001; Long, Agrawal & Rowe, 2012; Connallon & Clark, 2014; Rostant *et al*., 2015; Ruzicka *et al*., 2019). When a population faces conditions to which it has not been adapted, both sexes are displaced far from their evolutionary optima, which should, in turn, align selection in males and females (Fig. 2). On the other hand, long‐lasting evolution in ancestral environments might facilitate some degree of conflict resolution (Bonduriansky & Chenoweth, 2009), which should make the conflict less pronounced in ancestral *versus* novel environments with no historical selection to relax the conflict (see Delcourt, Blows & Rundle, 2009). Moreover, environmental heterogeneity and temporal variation make predicting conflict intensity even more challenging.

Besides its influence on male and female optimal values of traits (and hence the strength of selection on the sexes), the environment may impact IASC through modifying the genetic architecture of traits (Fig. 1). If environment modifies the strength of genetic correlations between the sexes, the intensity of IASC may change even in the absence of between‐environment variation in selection. IASC intensity is often measured as the intersexual genetic correlation for fitness (r_MF_), which is the fraction of sexually antagonistic genetic variance (between‐sex genetic covariance for fitness) relative to total additive genetic variance for fitness (see Table 1). It has been shown that both intersexual genetic covariance (Lyons, Miller & Meagher, 1994; Falconer & Mackay, 1996; Simons & Roff, 1996; Leips & Mackay, 2000; Vieira *et al*., 2000; Fox *et al*., 2004; Delcourt *et al*., 2009; Poissant *et al*., 2010; Punzalan, Delcourt & Rundle, 2014) and genetic variance (Via & Lande, 1987; Fowler & Whitlock, 2002; Charmantier & Garant, 2005) can be shaped by environment. However, environmental differences in these parameters are hard to predict. For example, Holloway, Povey & Sibly (1990) hypothesized that additive genetic variance should increase in novel environments because of the expression of genes that have not been selected in an ancestral environment, but multiple studies are equivocal whether less favourable and novel conditions cause a decrease or increase in additive genetic variance (e.g. Gebhardt‐Henrich & Van Noordwijk, 1991; Merilä, 1997; Merilä & Fry, 1998; Sgrò & Hoffmann, 1998, 2004; Hoffmann & Merilä, 1999; Merilä & Sheldon, 2001; Fowler & Whitlock, 2002; Charmantier & Garant, 2005).

Between‐environment changes in genetic architecture may result in low correlations within the same traits across different environments (Falconer & Mackay, 1996). Such a ‘breakdown of genetic architecture’ has gained solid empirical support (e.g. Simons & Roff, 1996; Rutherford & Lindquist, 1998; Bubliy, Loeschcke & Imasheva, 2001; Laugen, Laurila & Merilä, 2002, 2003*a*; Laugen *et al*., 2003*b*; Messina & Fry, 2003; Räsänen, Laurila & Merilä, 2003). Fewer studies, however, looked at sex specificity of changes in genetic architecture as a response to environmental factors (Guntrip, Sibly & Holloway, 1997). Understanding how male and female genetic variance and their covariance change between environments allows for assessing the adaptive potential of populations facing environmental change, while ignoring sex‐specific and between‐sex effects can lead to incorrect predictions about adaptive trajectories (Koch, Sbilordo & Guillaume, 2020).

Only by combining knowledge on how environment shapes the architecture of traits under selection and sex‐specific selection patterns, will we be able to predict IASC patterns and the probability that a population will adapt to a given environmental change as well as adaptation rate and trajectory. For example, Connallon & Hall (2016) modelled sex‐specific adaptation under different types of environmental change and showed that whether selection in both sexes aligns or conflicts depends on the genetic architecture of traits of interest as well as on the sensitivities of male and female optima to environmental change. It is also dependent on the dynamics of environmental change, i.e. whether environment factors change continuously (e.g. as in the case of climate warming or other human‐induced changes) or cycle over time (which can be observed in biotic conflicts such as host–pathogen interactions), and on how fast these changes are. More specifically, IASC is predicted to occur under directional (continuous) environmental change or slow environmental cycles, especially if genetic correlations between the sexes are strong. On the contrary, rapid cycles of environmental change or environmental change that shifts optima for male and female traits in similar directions are expected to align selection between the sexes.

### (2) Testing theory: experimentally manipulated environments

Much effort in empirical studies has been made to verify if novel/stressful environments align selection in the sexes and reduce IASC, with almost no empirical attempts to assess the role of cycling environmental changes on IASC. Most studies measured intersexual genetic correlations for fitness (r_MF_) in two or more environments, but other designs have also been incorporated (see online Supporting information, Table S1). Some studies supported the idea that IASC is most pronounced in environments to which a population is adapted and declines in a novel environment and/or under stress (Long *et al*., 2012; Punzalan *et al*., 2014; Han & Dingemanse, 2017), while others found similar levels of IASC across environments or even increased conflict in stressful environments (Delcourt *et al*., 2009; Delph *et al*., 2011*a*; Martinossi‐Allibert, Arnqvist & Berger, 2017; Koch *et al*., 2020; see also Skwierzyńska, Radwan & Plesnar‐Bielak, 2018). The reason for these discrepancies is intriguing, given that they can be found even when the same species and environmental modifications are used [compare Delcourt *et al*. (2009) with Punzalan *et al*. (2014) in Table S1]. One possible explanation could be that the effects of ecological conditions on IASC depend on genetic background, and hence vary between populations of different evolutionary histories (see Connallon & Hall, 2016). For example, Berger *et al*. (2014) tested the dynamics of IASC at benign and stressful temperatures in isofemale lines originating from two distinct natural populations of the seed beetle *Callosobruchus maculatus* and showed high IASC in benign conditions and its reduction under stress in one of the populations, but low IASC levels at both temperatures in another population. This can be interpreted as an indication that the level of IASC and ecological impacts on it might differ not only among species, but also among populations that vary in their sex‐specific genetic architecture of traits. Testing this would require comparing how IASC changes with environmental manipulations in populations differing in genetic architecture. This can be achieved either by testing populations shown to differ in sex‐specific genetic variances/cross‐sex covariances for traits of interest or by artificially creating populations varying in their genetic architecture.

### (3) Novel environments and stressful environments

While novel environments are usually considered stressful, the actual nature and amount of stress might be greatly dependent on a given environmental factor. Some novel environments will impose great stress on a population, but in others stress might be mild or even absent. In addition, the intensity of IASC might depend on the properties of a given environment rather than simply on its stressfulness. To our knowledge, there have only been three studies that systematically assessed the generality of IASC patterns by testing the intensity of sexual conflict and associated gender load in a set of novel environments (see Table S1). One of them showed that intersexual genetic correlation for fitness in *Tribolium castaneum* was positive (no IASC) in the non‐stressed control treatment. While it decreased in drought, heat and a treatment combining drought and heat, a (non‐significantly) negative value (indicating IASC) was observed only under heat stress (Koch *et al*., 2020). Intersexual genetic correlations for fitness in *Drosophila serrata* were shown to vary substantially on a variety of food media, ranging from negative values indicating the existence of conflict, to positive ones, suggesting no conflict, in these environments (Punzalan *et al*., 2014). Similarly, Skwierzyńska *et al*. (2018) demonstrated costs for females carrying genes associated with the expression of a male‐limited sexually selected trait (thickened legs used as a weapon and the associated aggressive strategy of ‘fighter’ male phenotype; see Appendix S1) in the bulb mite *Rhizoglyphus robini* at high, stressful temperature, but not at low temperature. This low temperature, while novel to the animals tested, is probably associated with milder stress. However, the authors suspected that the observed pattern might be specific to temperature itself. This is because the metabolic costs of expressing a fighter strategy (enlarged legs and fighting) should be high and sensitive to temperature. Genes affecting metabolism in fighter males are likely to have pleiotropic effects on females so that their costs are likely paid not only by males, but also by females, even though females do not express enlarged legs. This scenario seems probable, given that benefits of expressing enlarged legs and the fighter strategy in males have indeed been shown to vary with temperature (Plesnar‐Bielak *et al*., 2018). In general, while the above studies show that the intensity of IASC and associated costs to females can vary between different novel environments, more work is needed to uncover if this variation is related to the level of stress imposed by a given environment or depends on other properties of the environment. For example, a study incorporating factorial design with different stressors of varying intensity would be very informative. In such a study, IASC under different conditions that generate similar levels of stress could be contrasted with IASC variation among different levels of a given stressor. In addition, more attention should be given to investigating IASC responses to physiological (non‐stressful) levels of variation in environmental factors.

### (4) Sexual conflict in the wild

Most of the data on ecological effects on IASC come from laboratory studies on model organisms (see Table S1). While such an approach allows careful control of experimental conditions of interest, the complexity of natural environments, with spatial and temporal variation of many interacting environmental variables, may generate IASC patterns that differ from those observed in laboratories. For example, laboratory studies have tested pure effects of temperature on IASC (Berger *et al*., 2014; Skwierzyńska *et al*., 2018), but variance in temperature is usually correlated with other climatic factors like relative humidity, water availability etc., that together may influence IASC. In addition, these climatic factors often vary temporally, in contrast to stable laboratory conditions. Although our knowledge on IASC patterns in nature is still very limited, there are studies that try to fill this gap. In a recent meta‐analysis, De Lisle *et al*. (2018) analysed microclimatic drivers of selection on over 700 sex‐specific estimates of selection in the wild (obtained from Siepielski *et al*., 2018) and found that sexually antagonistic selection was driven by temperature, precipitation and evaporative potential and was less intense in more extreme environments. Selection was also more sex‐concordant (IASC was lower) at high latitudes, characterized by less stable environmental conditions. The authors also suggested that environment often modifies the strength of sex‐specific selection without reversing its sign, confirming earlier findings that the direction of selection is relatively stable across environments (Morrissey & Hadfield, 2012).

In another study that investigated the ecology of IASC under natural conditions, Delph *et al*. (2011*a*) compared viability selection on flower and leaf traits in four populations of *Silene latifolia* that differed in water availability. They found IASC for leaf thickness in water‐limited populations, but not in those experiencing relatively high rainfall. In water‐stressed populations, males that had thicker leaves survived better, but females in these populations were under stabilizing selection or weak negative selection for leaf thickness. This can be explained by the fact that males generally have thinner leaves and higher rates of leaf gas exchange than females (Delph, 2007; Delph *et al*., 2010), making male individuals with the thinnest leaves more sensitive to low water availability. Importantly, the study quantified only viability selection, but not fecundity or sexual selection, which are likely to interact with viability selection and to differ between the sexes. Indeed, leaf thickness is negatively genetically correlated with the number of flowers produced by a plant (Delph *et al*., 2004, 2005, 2011*b*), with likely effects on fecundity and siring success, suggesting that the combination of relationships between viability, fecundity and sexual selection can be sensitive to environmental factors.

While the above studies constitute a good starting point, understanding relationships between different environmental factors and IASC patterns requires more studies to be conducted in natural or seminatural settings. In particular, studies investigating environmental factors other than those related to climate/microclimate (e.g. food quality, quantity, or availability) or a combination of climatic and other abiotic/biotic factors in this context are missing.

## III. INTERLOCUS SEXUAL CONFLICT AND ENVIRONMENT

### (1) Theory – direct and indirect environmental influences on interlocus sexual conflict

IESC arises when there is a difference in optimal outcome of male–female interactions between the sexes (Parker, 1979; Arnqvist & Rowe, 2005) and applies to traits related to courtship, mating frequency, fertilization, parental care, brood size etc. (Chapman *et al*., 2003). IESC may drive evolutionary changes *via* sexually antagonistic coevolution (SAC; see Table 1), where each attempt to reach an optimum for a particular trait by one sex results in costs to the other sex (Parker, 1979; Rice, 1998; Arnqvist & Rowe, 2005; Hosken, Archer & Mank, 2019). These costs (termed harm) can drive evolution of counteradaptations (resistance traits), which in turn impose costs on the harming sex and fuel further evolution of traits to overcome the defences of the other sex (persistence traits), usually resulting in an evolutionary arms race between the sexes (Parker, 1979; Holland & Rice, 1998; Hosken *et al*., 2019; see Table 1), although other evolutionary dynamics of IESC have also been described (see Rowe, Cameron & Day, 2005).

**Fig 3:**
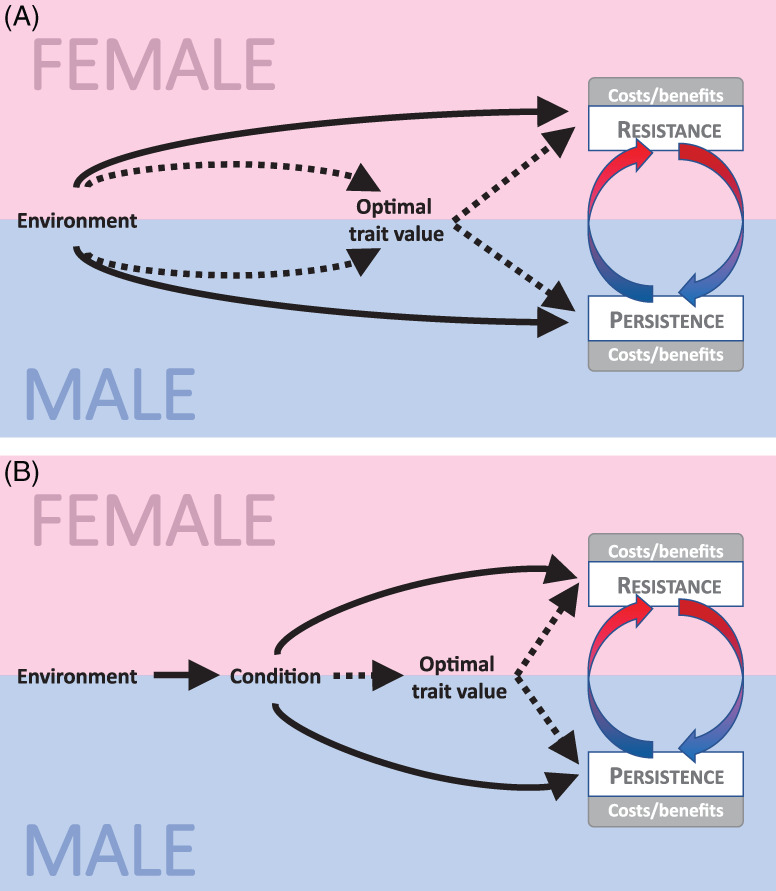
Schematic representation of how an environmental variable can modify interlocus sexual conflict (IESC) either directly (A) or indirectly through modifying condition (B), by affecting optimal value of the trait under conflict (dotted arrows) and/or cost to benefit ratio of resistance/persistence traits (solid arrows).

It has been demonstrated that costs and benefits of expressing traits mediating IESC may depend on ecological factors, such as resources, population density, mate availability, species composition, etc. (Emlen & Oring, 1977; Candolin & Heuschele, 2008; Fricke *et al*., 2009) (Table S2; Fig. 3A, solid arrows). As a consequence, when the strength and direction of natural selection on these traits differ between environments, male persistence and/or female resistance traits evolve in association with the environment (Arbuthnott *et al*., 2014*b*). Local environment may also affect the optimum of a trait under conflict (e.g. mating rate) for one or both sexes (Fig. 3A, dotted arrows) and change the relationship between traits mediating conflict (persistence and/or resistance traits) and a trait under conflict (Perry & Rowe, 2018).

Apart from direct effects on antagonistic traits themselves, environmental factors may affect condition of individuals (Fig. 3B). This in turn can determine the effectiveness of moving by each sex towards optimal values of traits under conflict (Fig. 3B, dotted arrows), as well as the costs and benefits of persistence and/or resistance traits (Fig. 3B, solid arrows). Based on condition‐dependence model, high‐condition individuals are expected to have higher benefits from elevated investment in costly sexual traits (Rowe & Houle, 1996; Tomkins *et al*., 2004). Thus, in cases where the costly sexual trait is associated with harmfulness, high‐condition males are predicted to harm females more (Friberg & Arnqvist, 2003). On the other hand, the same amount of male harm might be less detrimental to high‐condition females compared to those in poor condition, relaxing selection on high‐condition female resistance and decreasing IESC and SAC intensity. However, empirical studies on the seed beetle *Callosobruchus maculatus* (Iglesias‐Carrasco *et al*., 2018) and mite *Sancassania berlesei* (Łukasiewicz, 2020) do not seem to support the latter claim.

Another layer of complication comes from a bias in mate preferences. Under male mate preferences, females in better condition, and thus with greater reproductive potential, might receive more harmful courtship, a phenomenon called ‘the cost of being an attractive female' (Long *et al*., 2009, p. 3). Environment might influence this cost in two ways. First, it may affect variance in female condition and thus differences among females in experiencing male harm. Second, factors affecting social environment (e.g. sex ratio, population density) may modify the amount of harm imposed on high‐condition females as well as the strength of male preference.

Overall, local environment and its influence on individual condition may impact both the outcome of SAC and sexual conflict dynamics through many paths and mechanisms so that relationships between sexual conflict, SAC and ecology are complex. This complexity moves us away from full understanding of the dynamics of sexual conflict at both the micro‐ and macroecological scales (Miller & Svensson, 2014).

### (2) Plastic responses to environmental changes and their role in sexual conflict dynamics

The key to understanding of dynamics of IESC can be insight into what factors shape phenotypic plasticity in sexually selected traits, including those involved in sexual conflict. Most of the data regarding this topic comes from studies that used diet manipulations. Such manipulations affect individual condition, and, in consequence influence traits that mediate sexual conflict, such as ejaculate and seminal fluid protein composition in *Drosophila melanogaster* (Perry, Sirot & Wigby, 2013) or gustatory appeal of nuptial gifts in decorated cricket *Gryllodes sigillatus* (Rapkin *et al*., 2016). Female fitness consequences of sexual interactions have also been shown to depend on condition, especially after mating with more harmful male phenotypes (Pitnick & García‐González, 2002; Friberg & Arnqvist, 2003). For example, food quantity has been shown to affect mating behaviour in ladybird beetles (*Adalia bipunctata*) so that food‐deprived females remate less frequently and bias mating towards large, high‐condition males (Perry, Sharpe & Rowe, 2009). Similarly, the effect of sex peptide (see Appendix S1) on fecundity, lifespan and receptivity of *D. melanogaster* females depends on the quality of their diet (Fricke *et al*., 2010*b*).

While plastic responses of IESC‐mediating traits to resource quality/quantity manipulations have gained solid experimental support, plasticity in these traits in response to other environmental factors is relatively less explored. One promising avenue of research could be investigating plastic responses of sexual conflict‐related traits along an axis of more‐ *versus* less‐stressful environments. For example, the level of male‐induced harm in *D. melanogaster* is plastically reduced in both colder and hotter environments compared to intermediate ones (García‐Roa, Chirinos & Carazo, 2019), suggesting that plasticity of sexual behaviour can limit the negative impact of IESC on viability of populations experiencing rapid climate change. Future research will need to explore the patterns of plastic responses in traits involved in sexual conflict in detail, for example by comparing plasticity after exposure to environmental change at different life stages or examining fluctuating and spatially heterogeneous environments. Such studies should also consider impacts of plasticity in IESC‐related traits on population viability. Furthermore, the response of plastic traits involved in sexual conflict to selection is an intriguing area for future study.

Importantly, since IESC dynamics concern traits in both sexes, determining how environment affects sexual conflict requires simultaneous measurement of both male and female conflict traits (Table S2). This is illustrated by a study in which decreased quality of juvenile diet had no impact on male harm or female resistance to this harm in *S. berlesei* mites, despite a clear negative effect on male competitive abilities (Łukasiewicz, 2020). Moreover, it has been shown that different traits can respond to environmental manipulation in different directions (Fricke *et al*., 2010*b*), illustrating that any conclusions drawn from measuring only one fitness component are incomplete and may even be misleading. Investigating resistance and harm in their full complexity is thus important in future research.

### (3) IESC in populations adapted to different environments

Environmental influences on IESC are not limited to inducing plasticity in persistence and resistance traits, but also involve evolutionary responses in conflict‐related traits. While isolated populations undergoing SAC may accumulate divergent adaptations to sexual selection and evolve divergence even in the absence of environmental differences between these populations (Parker & Partridge, 1998; Panhuis *et al*., 2001; Gavrilets & Hayashi, 2005), differentiated ecological conditions may significantly affect this process (Arbuthnott *et al*., 2014*b*). An important line of evidence for the impact of ecological factors on IESC and its role in shaping population divergence comes from experimental evolution studies where populations adapted to new environments. For example, Fricke, Andersson & Arnqvist (2010*a*) found less divergence in both male harm and female resistance between seed beetle populations under stronger directional selection imposed by a novel food environment. Arbuthnott *et al*. (2014*b*) showed that in *D. melanogaster* both male effects on female longevity and female resistance can be affected by the particular environment to which a population is adapted, confirming ecologically dependent parallel evolution of male‐induced harm and female resistance. On the other hand, divergence of other traits linked to male competitiveness was not associated with adaptation to different environments (Arbuthnott, Agrawal & Rundle, 2014*a*).

The above results indicate that sexual conflict as an engine of divergence and speciation should not be considered as ecology independent. While sexual conflict might in fact drive divergence in the absence of ecological differences between populations (Martin & Hosken, 2003), many empirical studies suggest that sexual conflict alone may not be a universal ‘engine of speciation’ (Wigby & Chapman, 2006; Bacigalupe *et al*., 2007; Gay *et al*., 2009; Plesnar‐Bielak *et al*., 2013). However, ecological factors can interact with sexual conflict, reinforcing or weakening its potential to fuel speciation.

### (4) Sexual conflict in simple laboratory conditions and natural environments

Awareness is growing that simplified laboratory conditions may bias our understanding of the evolutionary consequences of sexual conflict. Environmental complexity or physical structure affects resource availability, predation risk, population density, availability of refuges, etc., which in turn can modify sexual conflict mode and/or SAC intensity (Rowe, 1992; Krupa & Sih, 1993; Magurran & Seghers, 1994; Rowe *et al*., 1994; Eldakar *et al*., 2009; Gosden & Svensson, 2009; Perry & Rowe, 2018).

The significance of this phenomenon was recently emphasized by a set of experiments comparing intersexual interactions of *D. melanogaster* in different physical environments – small and simple *versus* big and more complex (Yun *et al*., 2017; MacPherson *et al*., 2018; Malek & Long, 2019). The use of more‐complex environments, which provided shelters for females, decreased male‐induced harm to females and reduced the frequency of sexual interactions (Yun *et al*., 2017), thus increasing female fecundity (Malek & Long, 2019). In addition, while sexual interactions in *D. melanogaster* are biased towards high‐quality females in simple environments, no such bias was observed when a more complex environment was used (Yun *et al*., 2017). As a consequence, the ‘costs of being an attractive female’ are reduced in more‐complex environments. Additionally, the effect of male harm has been shown to be more detrimental for low‐ *versus* high‐quality females in complex but not in simple environments (MacPherson *et al*., 2018).

Similar results have been found in water striders *Aquarius remiges*, where a spatially structured environment that provided the possibility for free dispersal among groups of individuals reduced sexual conflict (Eldakar *et al*., 2009). In this study, females actively moved away from aggressive males, affecting costs and benefits of male aggression. In consequence, the more complex environment relaxed the correlation between male harm/aggression and mating success.

It is important to note, however, that the ‘complex’ and ‘structured’ environments used in the above studies are still far less spatially heterogeneous than those used by fruit flies or water striders in their natural habitats. The fact that providing animals with a slightly more natural environment compared to what is typically used in experimental studies resulted in considerable relaxation of sexual conflict suggests that the role of sexual conflict might in fact be overestimated in laboratories. Moving our experiments to more natural settings is therefore important for a more realistic understanding of sexual selection and conflict. Investigating conflict patterns in populations coming from their natural environment (in opposition to those evolved in the laboratory) should also provide new insights into our understanding of sexual conflict.

Another important simplification that differentiates natural and laboratory environments is that in the latter there are usually no interactions with other species. Environmentally induced changes in species composition may have indirect but substantial influence on IESC. They can substantially influence competition or predation risk, affecting the intensity of mating harassment (Magurran & Seghers, 1994; Gomez‐Llano, Bensch & Svensson, 2018). For example, it has been shown that male harassment in seaweed flies (*Coelopa* sp.) may depend on the relative proportion of brown algae within wrack beds, because different algae species stimulate male harm with varying intensity (Edward & Gilburn, 2007). This shows that interactions within natural communities may have important consequences on the relationship between IESC and environment and emphasizes the importance of investigating sexual conflict in natural conditions.

## IV. ENVIRONMENTAL SENSITIVITY OF SEXUAL CONFLICT – CONSEQUENCES FOR LOCAL ADAPTATION

### (1) Intralocus sexual conflict

While the potential role of IASC in adaptation has been considered both theoretically and empirically (reviewed in Bounduriansky & Chenoweth, 2009), environmental change has traditionally been assumed to influence both sexes symmetrically. The fact that a novel environment may affect fitness in a sex‐specific manner has been largely overlooked when investigating sexual conflict in the context of adaptation.

Notable exceptions are studies on desiccation resistance (Chippindale *et al*., 1998; Kwan *et al*., 2008) and thermal adaptation (Hsu *et al*., 2020) in *D. melanogaster*. In the former two studies, the authors showed that pre‐existing sexual dimorphism in desiccation resistance in this species (males more vulnerable to dehydration) resulted in different life‐history strategies favoured in males and females. Selection for early maturation and mating in males conflicted with survival and selection for late reproduction in females. These differences resulted in divergent selection on resource management and developmental pathways, including traits that are likely genetically correlated between the sexes (Reeve & Fairbairn, 1996, 1999). Thus, sexual conflict introduced or reinforced by a novel environment may constrain adaptive responses in the sexes when between‐sex genetic correlations hamper sex‐specific divergence of traits and limit resolution of sexual conflict (Kwan *et al*., 2008).

IASC can also influence adaptation indirectly, by shaping genetic architecture and affecting population demography. IASC has the potential to maintain standing genetic variation (Rice & Chippindale, 2001; Connallon & Clark, 2014; Rostant *et al*., 2015) as it makes selection fluctuate: different alleles are selected depending on the sex in which they are expressed. In accordance with this, a recent influential study on *Drosophila* suggested that at least some antagonistic alleles may persist in populations over surprisingly long evolutionary timescales, with balancing selection on these alleles shaping long‐lasting genome‐wide patterns of genetic variation (Ruzicka *et al*., 2019). If genes under IASC influence performance under novel conditions, genetic variation maintained by IASC in ancestral environments could become useful in the initial stages of adaptation to an environmental change. However, IASC may also play a role in the maintenance of alleles that reduce population growth and can even lead to extinction (de Vries & Caswell, 2019). Moreover, if gender load resulting from IASC persists or is intensified by an environmental change, it can decrease mean fitness in an adapting population, influencing population demography and decreasing adaptation potential. On the other hand, under the latter scenario, heightened costs of sexual conflict may also reverse selection on conflict traits before a population is driven into extinction.

One factor of potentially great importance for determining the fate of an adapting population is the relationship between population spatial structure and demography. A recent model shows that when there is variation in how different patches contribute to the gene pool (hard selection) and local population productivity depends more on females than on males (a common phenomenon called ‘female demographic dominance’; Crowley, 2000), selection will favour female‐beneficial alleles over male‐beneficial ones (Harts, Schwanz & Kokko, 2014). This might improve global performance of a population, but may also make the population unable to persist in some habitat patches by hampering adaptation in males (Harts *et al*., 2014). Thus, predicting the role of IASC in adaptation requires a deep understanding of the system of interest including the associations between IASC, genetic architecture, population structure, demography and viability (see also Alpedrinha *et al*., 2019).

### (2) Interlocus sexual conflict

The existence of gender load can result not only from IASC but also IESC. This is because the latter imposes costs on female fecundity/longevity and negatively impacts population demography. In this way sexual conflict can reduce population viability and, in extreme cases, lead to its extinction (‘the tragedy of the commons'; Rankin, Bargum & Kokko, 2007; see Table 1). On the other hand, male harm may not influence population demography as long as the population produces many more offspring than can survive to reproduction. However, under environmental change, mortality can be elevated and death rate can exceed birth rate, unmasking the effect of male harm on population viability (Holman & Kokko, 2013). In this way negative impacts of sexual conflict on population demography can counterbalance benefits of sexual selection for populations facing environmental challenge.

This effect may be exacerbated or reduced by changes in IESC imposed by environmental shifts. For example, temperature variation modulates male harm in *D. melanogaster* so that the impact of sexual conflict on population productivity is reduced in more extreme conditions (García‐Roa *et al*., 2019). The fact that this pattern results from behavioural plasticity suggests that sudden environmental changes have the potential to impose rapid shifts in sexual conflict dynamics and balance between costs (IESC) and benefits of sexual selection. Moreover, a recent study on seed beetles suggests that gender load associated with IESC may be reduced under environmental change (Martinossi‐Allibert *et al*., 2018). When populations evolved under different mating systems were subjected to stressfully elevated temperature, costs of intersexual and intrasexual interactions tended to decrease in polygamous and monogamous populations, but not in populations in which the evolution was limited to males (so that females were prohibited from coevolving with males). However, population fitness was equally affected by the stressful environment in all three mating regimes, suggesting that it is governed by an interplay between gender load, good genes effects and sex‐specific environmental robustness.

## V. LINKING INTRA‐ AND INTERLOCUS SEXUAL CONFLICT IN AN ENVIRONMENTAL CONTEXT: OUTSTANDING QUESTIONS

### (1) How does ecology shape interplay between the two types of conflict and their relative importance?

Both intra‐ and interlocus sexual conflict can operate in the same population (see Berger *et al*., 2016). They can involve different traits or both conflict types may act on one trait. For example, Berger *et al*. (2016) showed that in seed beetle populations, IASC lowering female fecundity can act simultaneously with IESC operating *via* male‐induced harm and reduce population productivity below the level expected if just one of the conflict types was present.

Both IASC and IESC might be affected by environmental conditions, and this effect might be more pronounced in relation to one or the other type of conflict. For example, it seems that decreased temperature removes IASC over the expression of genes associated with male morphs in the bulb mite (Plesnar‐Bielak *et al*., 2018; Skwierzyńska *et al*., 2018). At the same time, it reduces the selective advantage of the *6Pdgh* allele associated with increased male competitiveness and lowered fitness of females mating with males possessing this allele (Plesnar‐Bielak, Skwierzyńska & Radwan, 2020; see Appendix S1), thus also relaxing IECS. Theoretically, the same environmental change can even relax IASC and magnify IESC or *vice versa* so that the relative intensity of IASC and IESC might depend on ecological conditions. On the other hand, if both forms of conflict are intensified in a given novel environment, gender load is considerably increased, which can impede adaptation or even lead to extinction. Despite these potentially important consequences, the environmental sensitivity of both conflict types has, to our knowledge, never been investigated at the same time in a single population.

### (2) Are our laboratory estimates of conflict prevalence correct?

Improving our understanding of sexual conflict ecology is necessary to bridge the gap between laboratory estimates of occurrence and magnitude of sexual conflict and what happens in the wild. As discussed above, IESC might be more pronounced in over‐simplistic laboratory environments compared to what could be observed in nature (Yun *et al*., 2017; Malek & Long, 2019). In addition, if IASC is reduced in harsh, stressful environments, its prevalence may be overestimated by laboratory studies that have traditionally been conducted in benign environments to which study populations had been adapted (Long *et al*., 2012; Connallon & Hall, 2016). Moreover, environmental conditions in the vast majority of natural habitats are heterogeneous and/or variable in time, while laboratory studies are usually conducted in stable environments with hardly changing conditions. This may lead to further mismatches between the levels of conflict assessed in laboratories and those that shape the evolution of natural populations. While awareness of this problem is growing, much more work is needed to understand the extent to which results obtained in laboratories mirror the prevalence and importance of sexual conflict in various naturally occurring evolutionary processes. It is thus time to introduce more natural settings into our laboratories, and equally to put more effort into investigations of sexual conflict and its dynamics in the wild.

### (3) What is the role of environmental cycles and fluctuations in shaping sexual conflict?

While studying the impact of different environmental variables on sexual conflict, researchers use treatments that differ in a given ecological variable. However, the levels of these variables have always been kept constant, while fluctuating and oscillating conditions are the norm in natural conditions. Thus, we lack data on how such fluctuations and oscillations affect conflict intensity and dynamics. What is more, we need to develop a solid theoretical framework (Connallon & Hall, 2016) that incorporates temporal and spatial variation into sexual conflict research, which could guide future experimental studies.

## VI. CONCLUSIONS

1. The ecology of sexual conflict is increasingly gaining the attention of evolutionary biologists (e.g. Fricke *et al*., 2009; Perry & Rowe, 2018; Svensson, 2019).
2. There are still many research areas in which substantial effort is needed to advance our understanding of the processes driving conflict patterns.
3. There is a huge gap in our understanding of how environmental fluctuations and oscillations affect sexual conflict.
4. Much work remains to be done concerning the interplay of intra‐ and interlocus sexual conflict in various environments and how they feed back into evolutionary processes.
5. Better knowledge on these important topics could help us understand the responses of populations and communities to anthropogenic environmental changes.
6. More attention should also be devoted to understanding sexual conflict in natural settings and wild populations.

## Supporting information


**Table S1**. Characteristics of studies showing the effect of changing environments on intralocus sexual conflict (IASC) dynamics.

**Table S2**. Characteristics of studies showing the effect of changing environments on interlocus sexual conflict (IESC) dynamics.

**Appendix S1**. Examples of sexual conflict and how it is affected by environment.
