# Supplementary material for: Sexual conflict in a changing environment
Source: Biol Rev Camb Philos Soc. 2021 May 7;96(5):1854–67. doi: 10.1111/brv.12728 (PMC8518779; doi:10.1111/brv.12728)
Supplement: Supplementary file 1 — Table S1. Characteristics of studies showing the effect of changing environments on intralocus sexual conflict (IASC) dynamics. [file BRV-96-1854-s003.docx]

**Table S1.** Characteristics of studies showing the effect of changing environments on intralocus sexual conflict (IASC) dynamics.

| **STUDY:** | Punzalan *et al*. (2014) | | |
| --- | --- | --- | --- |
| **SPECIES:** | *Drosophila serrata* | | |
| **ENVIRONMENT:**  **DESIGN:** | Different food media: yeast, cornflour, rice flour, standard yeast medium with salt  Measuring r_MF_^*^ using controlled crosses between inbred lines | | |
| **RESULTS** | | **IASC REDUCED BY STRESS?** | **OTHER CONCLUSIONS** |
| r_MF_ negative for corn environment with relatively high fitness: r_MF_ differed among environments (resulting from differences in the genetic variances of the fitness of both sexes | | Yes | r_MF_ depends on a given environment |
| **STUDY:** | Delcourt *et al*. (2009) | | |
| **SPECIES:** | *Drosophila serrata* | | |
| **ENVIRONMENT:**  **DESIGN:** | Different food media: yeast (ancestral) and corn (novel)  Measuring r_MF_ using half-sibling breeding design | | |
| **RESULTS** | | **IASC REDUCED BY STRESS?** | **OTHER CONCLUSIONS** |
| r_MF_ negative in both environments but not significantly different from 0 in corn. The major axis of genetic variation was IASC in both environments | | No | IASC in both environments, but stronger in novel one |
| **STUDY:** | Long *et al*. (2012) | | |
| **SPECIES:** | *Drosophila melanogaster* | | |
| **ENVIRONMENT:** | Cadmium in the media | | |
| **DESIGN** | Comparing effects of sexually successful *versus* unsuccessful males on fitness in adapted and non-adapted populations | | |
| **RESULTS** | | **IASC REDUCED BY STRESS?** | **OTHER CONCLUSIONS** |
| In adapted populations sexually successful males sired unfit daughters; in non-adapted populations attractive males sired high-fitness offspring | | Yes |  |
| **STUDY:** | Martinossi-Allibert *et al*. (2017) | | |
| **SPECIES:** | Bean beetle *Acanthoscelides obtectus* | | |
| **ENVIRONMENT:**  **DESIGN:** | Different host plants white bean (ancestral) and chickpea (novel)  Comaparing r_MF_ using isofemale lines in the two evolution regimes cross-reared on the alternative hosts | | |
| **RESULTS** | | **IASC REDUCED BY STRESS?** | **OTHER CONCLUSIONS** |
| r_MF_ low but positive; only one estimate in adapted population close to significantly different from 0 | | No |  |
| **STUDY:** | Han & Dingemanse (2017) | | |
| **SPECIES:** | Southern field crickets *Gryllus bimaculatus* | | |
| **ENVIRONMENT:**  **DESIGN:** | Free-choice and protein-deprived diets  Comparing r_MF_ for survival of crickets raised on the two diets | | |
| **RESULTS** | | **IASC REDUCED BY STRESS?** | **OTHER CONCLUSIONS** |
| Intersexual genetic covariance and correlation greater than 0 and not significantly different from 1 for protein-deficient diet; not significantly different from 0 but different from 1 in free-choice diet treatment | | Yes |  |
| **STUDY:** | Delph *et al*. (2011*a*) | | |
| **SPECIES:** | *Silene latifolia* | | |
| **ENVIRONMENT:**  **DESIGN:** | Water limitation  Measuring viability selection in populations from habitats with more or less rain | | |
| **RESULTS** | | **IASC REDUCED BY STRESS?** | **OTHER CONCLUSIONS** |
| Selection on thicker leaves in males, but only in water-limited populations; weakly negative selection or stabilizing selection on leaf thickness in females | | No | IASC in stressed population |
| **STUDY:** | Berger *et al*. (2014) | | |
| **SPECIES:** | Seed beetle *Callosobruchus maculatus* | | |
| **ENVIRONMENT:**  **DESIGN:** | Temperature: 29 °C (benign) and 36 °C (stressful)  Measuring r_MF_ using isofemale lines | | |
| **RESULTS** | | **IASC REDUCED BY STRESS?** | **OTHER CONCLUSIONS** |
| In one population substantial IASC under benign conditions and reduction of IASC under stress; in another population low IASC at both temperatures. Isofemale lines with male-beneficial and female-detrimental alleles at benign conditions had low fitness under stress | | Yes | IASC increases population’s susceptibility to stress.  Effects of stress on IASC differ among populations |
| **STUDY:** | Lankinen & Hydbom (2017) | | |
| **SPECIES:** | *Collinsia heterophylla* | | |
| **ENVIRONMENT:**  **DESIGN:** | Soil resource environment  Hand-pollinations and measuring conflict traits | | |
| **RESULTS** | | **IASC REDUCED BY STRESS?** | **OTHER CONCLUSIONS** |
| No universal effect of environment on conflict traits (time of stigma receptivity and seed production) | | No |  |
| **STUDY:** | Skwierzyńska *et al*. (2018) | | |
| **SPECIES:** | Bulb mite *Rhizoglyphus robini* | | |
| **ENVIRONMENT:**  **DESIGN:** | Temperature: 18 °C (novel, non- or mildly stressful), 24 °C (ancestral) or 28 °C (novel, stressful)  Comparing female fitness in lines selected for male sexual trait previously shown to be associated with IASC | | |
| **RESULTS** | | **IASC REDUCED BY STRESS?** | **OTHER CONCLUSIONS** |
| Females from lines without male sexual trait more fecund than females from lines with male sexual trait at 28 °C; the reverse pattern observed at 18 °C | | No | Female costs associated with IASC differ among novel environments |
| **STUDY:** | de Lisle *et al*. (2018) | | |
| **SPECIES:** | A range of species | | |
| **ENVIRONMENT:**  **DESIGN:** | Climatic factors  Meta-analyses of a large data set on environmental drivers of selection on a global scale | | |
| **RESULTS** | | **IASC REDUCED BY STRESS?** | **OTHER CONCLUSIONS** |
| IASC conflict reduced in extreme environments, but depended on the measure of IASC | | Yes |  |
| **STUDY:** | Koch *et al*. (2020) | | |
| **SPECIES:** | Red flour beetle *Tribolium castaneum* | | |
| **ENVIRONMENT:**  **DESIGN:** | Drought, heat, drought + heat  Comparing r_MF_ and other aspects of genetic architecture in different treatments | | |
| **RESULTS** | | **IASC REDUCED BY STRESS?** | **OTHER CONCLUSIONS** |
| Highest positive r_MF_ in control; lower under all stress treatments; and negative r_MF_ under heat stress | | No |  |

*r_MF_ = intersexual genetic correlation for fitness.
