# Supplementary material for: Sexual conflict in a changing environment
Source: Biol Rev Camb Philos Soc. 2021 May 7;96(5):1854–67. doi: 10.1111/brv.12728 (PMC8518779; doi:10.1111/brv.12728)
Supplement: Supplementary file 2 — Table S2. Characteristics of studies showing the effect of changing environments on interlocus sexual conflict (IESC) dynamics. [file BRV-96-1854-s001.docx]

**Table S2.** Characteristics of studies showing the effect of changing environments on interlocus sexual conflict (IESC) dynamics.

| **STUDY:** | Edward & Gilburn (2007) | |
| --- | --- | --- |
| **SPECIES:** | *Coelopa frigida* and *C. pilipes* | |
| **ENVIRONMENT:**  **DESIGN:**  **Conflict mediating traits measured in both sexes?** | Habitat composition – relative proportion of brown algae, *Fucus* and *Laminaria*  Comparing effect of habitat composition on male harassment towards females, defined as the proportion of males mounting a female  No, only in males | |
| **RESULTS** | | **OTHER CONCLUSIONS** |
| Level of male harassment by male *C. frigida* was stimulated more by *Laminaria* than *Fucus*; a similar effect was not observed in *C. pilipes* | |  |
| **STUDY:** | Eldakar *et al*. (2009) | |
| **SPECIES:** | *Aquarius remigis* | |
| **ENVIRONMENT:** | Single group (no dispersion) *versus* multi-group population (dispersion allowed) | |
| **DESIGN:**  **Conflict mediating traits measured in both sexes?** | Comparing male mating strategies and male mating success between dispersion treatments  No, only in males | |
| **RESULTS** | | **OTHER CONCLUSIONS** |
| Male aggression and harassment were positively correlated with mating success in no-dispersion environment. There was a quadratic relationship between male behaviour and mating when dispersal was permitted. Females moved away from male aggressors if dispersion allowed. | | Dispersion may affect costs and benefits associated with male aggression |
| **STUDY:** | Fricke *et al*. (2010*b*) | |
| **SPECIES:** | *Drosophila melanogaster* | |
| **ENVIRONMENT:**  **DESIGN:**  **Conflict mediating traits measured in both sexes?** | Different concentration of yeast in food during female adulthood  Comparing effect of sex peptide on female phenotypic traits  No, only in females | |
| **RESULTS** | | **OTHER CONCLUSIONS** |
| Exposure to sex peptide beneficial or costly to females depending on the diet regime | |  |
| **STUDY:** | Arbuthnott *et al*. (2014*a*) | |
| **SPECIES:** | *Drosophila melanogaster* | |
| **ENVIRONMENT:** | Food supplemented with either ethanol or cadmium chloride | |
| **DESIGN:**  **Conflict mediating traits measured in both sexes?** | Experimental evolution; comparing male-induced harm on female longevity and female resistance between populations adapted to different environments  Yes | |
| **RESULTS** | | **OTHER CONCLUSIONS** |
| Male-induced harm and female resistance evolve in association with adaptation to the different environments | |  |
| **STUDY:** | Rapkin *et al*. (2016) | |
| **SPECIES:** | *Gryllodes sigillatus* | |
| **ENVIRONMENT:**  **DESIGN:**  **Conflict mediating traits measured in both sexes?** | Different ratios of protein and carbohydrate intake  Comparing mass and amino acid composition of the nuptial gift and effect of nutrient intake on regulation of sexual conflict  No, only in males | |
| **RESULTS** | | **OTHER CONCLUSIONS** |
| Nuptial gift traits are maximized at high intake of nutrients. Males regulate their intake of nutrients at close to optimal nutrient ratios | | Balance of the intake of nutrients can affect the outcome of sexual conflict |
| **STUDY:** | Yun *et al*. (2017) | |
| **SPECIES:** | *D. melanogaster* | |
| **ENVIRONMENT:**  **DESIGN:**  **Conflict mediating traits measured in both sexes?** | Small and simple *versus* big and complex environment  Comparing male sexual behaviour, male-induced harm and male choice and its effect on selection on females  No, only in males | |
| **RESULTS** | | **OTHER CONCLUSIONS** |
| In the complex environment: (1) male harm was reduced (less sexual interactions); (2) male choice was no longer biased towards high-quality females; and (3) selection on females was strengthened | |  |
| **STUDY:** | Perry *et al*. (2017) | |
| **SPECIES:** | *Gerris incognitus* | |
| **ENVIRONMENT:** | A range of factors | |
| **DESIGN:**  **Conflict mediating traits measured in both sexes?** | Estimated the contributions of ecological context and sexual coevolution to the correlated evolution of antagonistic traits among 16 populations of water striders  Yes | |
| **RESULTS** | | **OTHER CONCLUSIONS** |
| Ecological variation for female spine, female body size divergence, and male pre-genital segment. Correlation between female and male traits involved in sexual conflict. | | Role of sexually antagonistic coevolution in driving population phenotypic divergence (see also Perry & Rowe, 2018) |
| **STUDY:** | MacPherson *et al*. (2018) | |
| **SPECIES:** | *Drosophila melanogaster* | |
| **ENVIRONMENT:** | Small and simple *versus* big and complex environment | |
| **DESIGN:**  **Conflict mediating traits measured in both sexes?** | Comparing effect of male-induced harm on low- and high-quality females  No, only in females | |
| **RESULTS** | | **OTHER CONCLUSIONS** |
| Effect of male-induced harm is greater for low-quality females than to high-quality females in the complex but not in the simple environment | | Male–male competition strengthened selection on females in the simple but not in the complex environment |
| **STUDY:** | Gomez-Llano *et al*. (2018) | |
| **SPECIES:** | *Calopteryx splendens* and *C. virgo* | |
| **ENVIRONMENT:** | Species composition and male density | |
| **DESIGN:**  **Conflict mediating traits measured in both sexes?** | Comparing effect of conspecific male density (high or low) and species composition (sympatric or allopatric) on male harassment and female survival  Yes | |
| **RESULTS** | | **OTHER CONCLUSIONS** |
| Interaction between male density and species composition strongly affects female survival – in low-density treatment, females survived better under sympatric compared to allopatric conditions. Interspecific interference competition reduced conspecific male mating success with large females. | | Interspecific competition might indirectly facilitate female survival by reducing mating harassment from conspecific males and thereby influence sexual conflict dynamics. |
| **STUDY:** | Iglesias-Carrasco *et al*. (2018) | |
| **SPECIES:** | *Callosobruchus maculatus* | |
| **ENVIRONMENT:** | Juvenile diet quality | |
| **DESIGN:**  **Conflict mediating traits measured in both sexes?** | Testing whether the costs of mating for females depend on her and/or male body condition in a 2 × 2 factorial design  No, only in females | |
| **RESULTS** | | **OTHER CONCLUSIONS** |
| (1) Females in better condition had higher reproductive output and lived longer, but (2) her mate’s body condition had no effect on female fitness; (3) no interaction between female and male condition for female fitness; (4) females that mated with males in better condition had shorter copulations and started to kick sooner. | |  |
| **STUDY:** | Malek & Long (2019) | |
| **SPECIES:** | *Drosophila melanogaster* | |
| **ENVIRONMENT:**  **DESIGN:**  **Conflict mediating traits measured in both sexes?** | Simple *versus* complex environment  Comparing direct and indirect female fitness between environments  No, only in females | |
| **RESULTS** | | **OTHER CONCLUSIONS** |
| Females remated less frequently and produced more offspring in complex than in simple environment; no differences in the fitness of sons or daughters of females housed in different environments | |  |
| **STUDY:** | García-Roa *et al*. (2019) | |
| **SPECIES:** | *Drosophila melanogaster* | |
| **ENVIRONMENT:**  **DESIGN:**  **Conflict mediating traits measured in both sexes?** | Temperature (±4°C)  Comparing effect of male-induced harm on female fitness and sexual behaviour of both sexes between regimes  Yes | |
| **RESULTS** | | **OTHER CONCLUSIONS** |
| Variations in environmental temperature modulate male harm *via* behavioural plasticity | |  |
| **STUDY:** | Łukasiewicz (2020) | |
| **SPECIES:** | *Sancassania berlesei* | |
| **ENVIRONMENT:**  **DESIGN:**  **Conflict mediating traits measured in both sexes?** | Juvenile diet quality  Examining condition dependence of male competitiveness, and intensity of sexual conflict  Yes | |
| **RESULTS** | | **OTHER CONCLUSIONS** |
| Juvenile diet quality affects male competitiveness but not male-induced harm nor female resistance to this harm | |  |
| **STUDY:** | Rostant *et al*. (2020) | |
| **SPECIES:** | *Drosophila. melanogaster* | |
| **ENVIRONMENT:**  **DESIGN:**  **Conflict mediating traits measured in both sexes?** | Different concentration of yeast in food  Experimental evolution; comparing two sexual conflict levels (male-biased or female-biased regimes) and two resource-level treatments (high or low yeast concentration)  No, only in females | |
| **RESULTS** | | **OTHER CONCLUSIONS** |
| In resource‐rich regimes, high‐conflict females evolved resistance to continual exposure to males, with no trade-offs with somatic maintenance, whereas in the poor resource treatment their ability to evolve resistance to males was compromised. | | Evolution of female resistance is dependent on resource level. |
