# Supplementary material for: Sexual conflict in a changing environment
Source: Biol Rev Camb Philos Soc. 2021 May 7;96(5):1854–67. doi: 10.1111/brv.12728 (PMC8518779; doi:10.1111/brv.12728)
Supplement: Supplementary file 3 — Appendix S1. Examples of sexual conflict and how it is affected by environment. [file BRV-96-1854-s002.docx]

**Appendix S1. Examples of sexual conflict and how it is affected by environment.**

**(1) Intralocus sexual conflict (IASC) – examples**

***Negative fitness correlations between the sexes in* Drosophila melanogaster**

In *Drosophila melanogaster* IASC has been studied extensively (e.g. Chippindale *et al.,* 2001; Long & Rice, 2007; Prasad *et al.*, 2007; Morrow, Stewart & Rice, 2008). Chippindale *et al.* (2001) measured intersexual genetic correlations for juvenile and adult fitness in a laboratory-adapted population. While juvenile survival was positively correlated between the sexes, the authors found a negative correlation for adult reproductive success, signifying IASC. In subsequent work, IASC in this species was found to be mediated by selection on locomotory activity, body size and development time (Long & Rice, 2007; Prasad *et al.*, 2007; Lund-Hansen, Abbott & Morrow, 2020). The level of IASC has been shown to depend on how well a population is adapted to its environment so that sexual antagonism dominates in well-adapted populations, while in non-adapted populations selection is more sex-congruent and sexual antagonism is reduced (Long, Agrawal & Rowe, 2012). Specifically, the authors compared the effect of male sexual attractiveness on their offspring fitness in cadmium-adapted populations and populations created by crossing cadmium-adapted populations with ethanol-adapted populations. Attractive, sexually successful males sired daughters of low fitness in adapted populations, while their offspring were of high fitness, regardless of sex, in maladapted populations.

***Male weaponry and the associated strategy of ‘fighter’ males in the bulb mite***

The bulb mite *Rhizoglyphus robini* is male dimorphic, with morphs differing in morphology and behaviour. More-aggressive ‘fighter’ males possessing enlarged third pair of legs, used to fight (and sometimes kill) rivals, can coexist with benign ‘scramblers’ with unmodified legs (Radwan & Klimas, 2001). In mixed-morph populations fighter males achieve higher reproductive success, and thus higher fitness, than scramblers. Because morphs are heritable, artificial selection on the frequencies of male phenotypes was used to show IASC associated with male morph expression (Plesnar Bielak *et al.*, 2014; see also Łukasiewicz, Niśkiewicz & Radwan, 2020). Selection for an increased proportion of fighters (high male fitness) resulted in a correlated decrease in female fecundity and longevity, indicating that genes associated with fighter expression have negative pleiotropic effects on female fitness, despite females not expressing enlarged legs. This result was obtained at 24°C, a temperature to which the mites had been adapted. Another study showed females from fighter-selected lines to be less fecund than females from the scrambler-selected lines at 28°C, but the reverse pattern at 18°C, indicating that female costs of expressing genes associated with fighter expression vary with temperature (Skwierzyńska, Radwan & Plesnar-Bielak, 2018).

**(2) Interlocus sexual conflict (IESC) – examples**

***Seminal fluids in* Drosophila**

Male ejaculates, containing not only sperm, but also seminal fluid proteins (SFPs) and other substances are extremely complex sexually selected phenotypes (Chapman, 2008; Avila *et al.*, 2012). For example, over 80 SFPs transferred to females while mating have been identified in *Drosophila melanogaster* (Swanson *et al.*, 2001). These ejaculate components, synthesized by the paired accessory glands, affect male competitiveness and manipulate female physiology and behaviour, stimulating egg production, reducing remating rate, and influencing sperm storage, feeding rate, food preference, and water balance (reviewed by Wolfner, 1997). Continuous exposure to sex peptide negatively affects female longevity and fitness (Wigby & Chapman, 2005). It has been shown that the scale of these costs, and their magnitude and sign, depend on food quality (Fricke, Bretman & Chapman, 2010*b*).

***Grasping and anti-grasping structures in* Gerris *spp.***

In many species of water striders repeated mating, forced by males, imposes fitness costs to females (Arnqvist & Rowe, 2002), resulting in sexual conflict over control of copulation. Thus, water strider males have evolved special grasping structures on pre-genital segments that help them to force copulation. Male grasping is essential for successful mating but causes harassment to females and is energetically costly to them (Rowe, 1994). It can also increase predation risk (Rowe, 1994). Dorsal spines, female anti-grasping structures, make females more resistant to male harassment and allow them control over mating decisions (Arnqvist & Rowe, 1995). Increased female resistance, however, reinforces sexually antagonistic selection favouring male grasping, resulting in spectacular struggles over mating. The role of ecological variation in shaping the coevolution of sexually antagonistic traits in water striders is recognized relatively well (Rowe *et al.*, 1994; Perry, Garroway & Rowe, 2017; Perry & Rowe, 2018), with climatic factors and physicochemical properties of water playing leading roles in determining IESC dynamics (Perry *et al.*, 2017).

***6Pgdh polymorphism in the bulb mite***

Allele polymorphism in the gene for 6-phosphogluconate dehydrogenase (*6Pgdh*) in the bulb mite is a striking example of a single gene involved in sexual conflict. 6Pgdh is part of the pentose phosphate cycle converting glucose and producing NADPH and ribulose-5-phosphate (Murray *et al.*, 2003). Males bearing the ‘winning’ *6Pgdh* allele (S) gain higher reproductive success than males lacking it (Konior, Radwan & Kolodziejczyk, 2001; Łukasik, Zygadło & Radwan, 2010), because they produce more sperm and copulate more frequently (Skwierzyńska & Plesnar-Bielak, 2018). At the same time, S-bearing males decrease the fecundity of their female partners (Konior *et al.*, 2006; Łukasik *et al.*, 2010; Skwierzyńska & Plesnar-Bielak, 2018), which is a clear indication of sexual conflict. The S allele advantage has been shown to depend on temperature and sex ratio (Plesnar-Bielak *et al.*, 2020), with selection favouring S being stronger in male-biased populations (with more intense sexual selection) and at higher temperature (24°C) and weaker under female-bias and at lower temperature (18°C). This shows that the dynamics of IESC associated with *6Pgdh* depends on environment.

.
